# Hereditary Hemochromatosis Type 2A Presenting With Hypogonadism, Diabetes, and Osteoporosis in a Young Woman

**DOI:** 10.1210/jcemcr/luaf148

**Published:** 2025-09-12

**Authors:** Ajeesh Thulaseedharan, Puthiyaveetil Khadar Jabbar, Sandra Mosses

**Affiliations:** Department of Endocrinology, Indian Institute of Diabetes, Thiruvananthapuram 695011, India; Department of Endocrinology, Indian Institute of Diabetes, Thiruvananthapuram 695011, India; Department of Endocrinology, Indian Institute of Diabetes, Thiruvananthapuram 695011, India

**Keywords:** Juvenile hemochromatosis, hypogonadotropic hypogonadism, diabetes mellitus, osteoporosis, iron overload

## Abstract

Hereditary hemochromatosis type 2A (juvenile hemochromatosis) is a rare autosomal recessive disorder caused by mutations in the *HJV* gene. It results in severe systemic iron overload and multiorgan dysfunction, including endocrine and skeletal manifestations. A 32-year-old woman presented with secondary amenorrhea, progressive skin hyperpigmentation, arthritis, and poorly controlled diabetes. Evaluation revealed hypogonadotropic hypogonadism, elevated serum ferritin (>2000 ng/mL), transferrin saturation of 93.8%, and osteoporosis (lumbar spine T-score −3.3; femoral neck −3.7). Magnetic resonance imaging showed iron deposition in the pituitary gland, liver, pancreas, and heart. Genetic testing confirmed a homozygous *HJV* mutation (c.1063G > T, p.Asp355Tyr). Management included weekly therapeutic phlebotomy (target ferritin ∼50 ng/mL), a basal-bolus insulin regimen, estrogen-progestin hormone replacement, and calcium/vitamin D supplementation. After 3 months, skin pigmentation lightened, glycated hemoglobin dropped from 8.5% to 7.8%, ferritin fell to 1294 ng/mL, and menstrual cycles resumed. This case emphasizes the importance of suspecting juvenile hemochromatosis in young patients with unexplained hypogonadism, diabetes, or osteoporosis. Early diagnosis and multidisciplinary treatment can prevent irreversible organ damage and improve long-term outcomes.

## Introduction

Hereditary hemochromatosis is an autosomal recessive disorder characterized by excessive intestinal iron absorption and systemic iron deposition, leading to multiorgan dysfunction [1]. Juvenile hemochromatosis (type 2A [JH]) is a rare aggressive variant caused by homozygous mutations in the *HJV* (hemojuvelin) gene or the *HAMP* (hepcidin antimicrobial peptide) gene [2]. It typically presents in the second or third decade with severe endocrine dysfunction, cardiomyopathy, and advanced liver fibrosis [3]. Endocrine manifestations such as hypogonadotropic hypogonadism and diabetes mellitus often occur early due to iron deposition in the pituitary and pancreas [3]. Bone disease (osteoporosis) is also common in iron overload, partly because of hypogonadism and direct iron toxicity [4].

Early diagnosis of JH is crucial because delayed treatment allows rapid iron accumulation and irreversible organ damage. Diagnosis requires a high index of suspicion in young patients with the classic triad of skin hyperpigmentation, diabetes, and hypogonadism. We report a case of *HJV*-related juvenile hemochromatosis in a 32-year-old woman who presented with secondary amenorrhea, poorly controlled diabetes, and severe osteoporosis. This case highlights the importance of recognizing this disorder and the need for comprehensive evaluation and management of endocrine and bone manifestations.

## Case Presentation

A 32-year-old woman presented with secondary amenorrhea for 5 years, poorly controlled diabetes mellitus for 3 years, and progressive skin hyperpigmentation. She also reported arthritis involving the right metacarpophalangeal joints and persistent fatigue. There was no family history of endocrine or hereditary disorders, and no history of alcohol use, blood transfusions, or chronic infections.

Her menstrual history was notable for menarche at age 13 years, followed by oligomenorrhea (cycles of 60-90 days). She had been managed with cyclic estrogen-progestin therapy in her mid-20s but remained amenorrheic for the past 5 years. She achieved a spontaneous conception within 1 year of marriage (age 24 years); her only childbirth was at age 24 years, 8 years before presentation. Diabetes mellitus was diagnosed 3 years earlier after polyuria, polydipsia, and weight loss; however, glycemic control remained suboptimal despite multiple insulin regimens.

**Figure 1.**
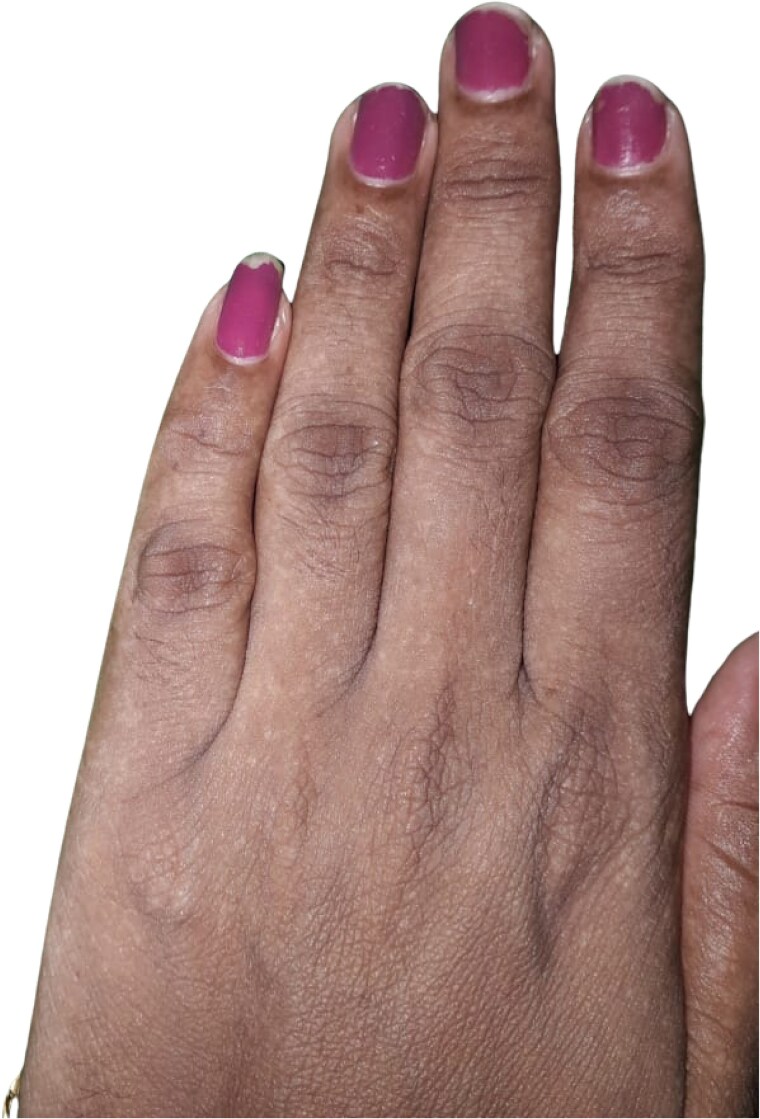
Skin hyperpigmentation on the dorsum of the hand, consistent with systemic iron overload.

On examination, her weight was 58.5 kg, and her height was 162 cm (body mass index 22.3 kg/m^2^). Vital signs were normal (blood pressure 118/72 mm Hg, pulse 78/min). Skin examination revealed generalized hyperpigmentation, particularly on the dorsum of the hands and feet (Fig. 1). There was mild swelling of the right metacarpophalangeal joint. Cardiovascular, respiratory, and abdominal examination were unremarkable: heart sounds were normal, and there was no hepatosplenomegaly. The neurological examination was also normal.

## Diagnostic Assessment

Laboratory investigations (Table 1) demonstrated *hypogonadotropic hypogonadism*: LH 0.58 mIU/mL (0.58 IU/L; normal follicular range 1.9-12.5 mIU/mL) and FSH 1.39 mIU/mL (1.39 IU/L; normal 1.9-12.5 mIU/mL), with an estradiol level of 11.6 pg/mL (42.7 pmol/L; normal 30-350 pg/mL). The morning cortisol and TSH were within normal limits (cortisol 12.4 µg/dL, TSH 2.37 mIU/mL). Markers of iron status showed severe overload: serum ferritin was >2000 ng/mL (>2.00 µg/L; reference 20-200 ng/mL) and serum iron 265.3 µg/dL (47.5 µmol/L; reference 50-170 µg/dL [9-30 µmol/L]). Transferrin saturation was 93.8% (ref. 15-45%). Liver enzymes were mildly elevated (AST 28 IU/L, ALT 32 IU/L; normal 0-40 IU/L for both). Serum calcium (9.2 mg/dL [2.30 mmol/L], ref. 8.5-10.5 mg/dL) and phosphate (3.5 mg/dL [1.13 mmol/L], ref. 2.5-4.5 mg/dL) were normal. 25-hydroxyvitamin D was low at 20 ng/mL (50 nmol/L; ref. 30-100 ng/mL). Parathyroid hormone was 45 pg/mL (4.8 pmol/L; ref. 10-65 pg/mL). Hemoglobin was 13.1 g/dL, with normal red blood cell indices (Mean Corpuscular Volume [MCV] 8 fL, Mean Corpuscular Hemoglobin [MCH] 28 pg). Her most recent HbA1c was 8.5% (69 mmol/mol; goal <7%).

**Table 1.** Laboratory investigations on presentation (hormones, iron studies, metabolic parameters)

Test	Patient value	Reference range
TSH	2.37 mIU/mL	0.4-4.5 mIU/mL
LH	0.58 mIU/mL (0.58 IU/L)	1.9-12.5 mIU/mL (1.9-12.5 IU/L)
FSH	1.39 mIU/mL (1.39 IU/L)	1.9-12.5 mIU/mL (1.9-12.5 IU/L)
Estradiol	11.6 pg/mL (42.7 pmol/L)	30-350 pg/mL (110-1280 pmol/L)
AST	28 IU/L	0-40 IU/L
ALT	32 IU/L	0-40 IU/L
Calcium	9.2 mg/dL (2.30 mmol/L)	8.5-10.5 mg/dL (2.12-2.62 mmol/L)
Phosphate	3.5 mg/dL (1.13 mmol/L)	2.5-4.5 mg/dL (0.81-1.45 mmol/L)
PTH	45 pg/mL (4.8 pmol/L)	10-65 pg/mL (1.07-7.0 pmol/L)
25(OH) Vitamin D	20 ng/mL (50 nmol/L)	30-100 ng/mL (75-250 nmol/L)
Ferritin	>2000 ng/mL (>2.00 mg/L)	20-200 ng/mL (0.02-0.20 mg/L)
Iron	265.3 µg/dL (47.5 µmol/L)	50-170 µg/dL (9-30 µmol/L)
Transferrin saturation	93.8%	15-45%
HbA1c	8.5% (69 mmol/mol)	<7% (<53 mmol/mol)

All values are reported with conventional and SI units at first mention.

Abbreviations: 25(OH) Vit D, 25-hydroxyvitamin D; ALT, alanine aminotransferase; AST, aspartate aminotransferase; HbA1c, glycated hemoglobin.

This table summarizes key laboratory investigations during patient presentation, including thyroid function, gonadotropins, estradiol, liver enzymes, bone metabolism markers, iron studies, and glycemic control. All results are reported in both conventional and SI units. Abnormal values include suppressed gonadotropins (LH, FSH), low estradiol, vitamin D deficiency, markedly elevated ferritin and transferrin saturation, and poor glycemic control (elevated HbA1c).

**Figure 2.**
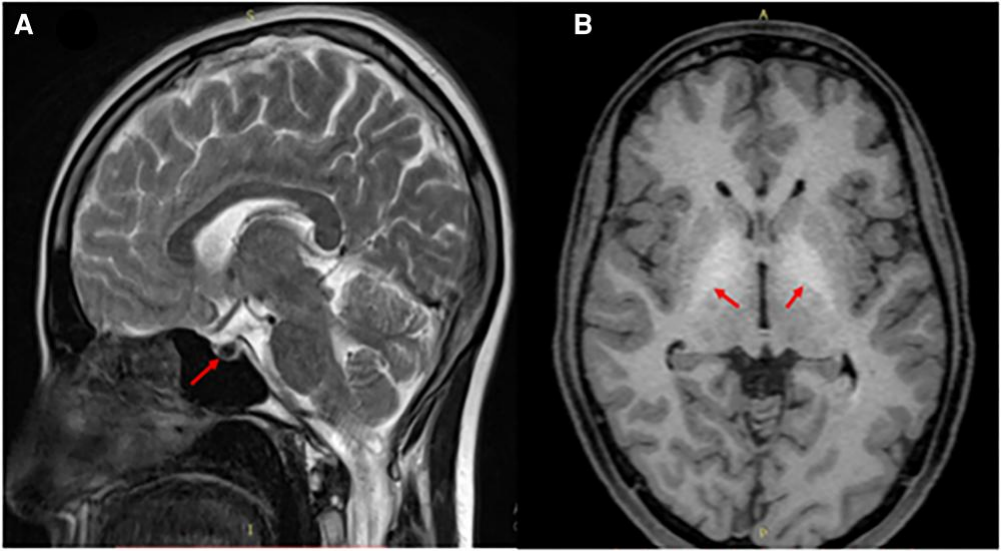
(A). T2-weighted MRI showing hypointensity of the pituitary gland, consistent with iron deposition. (B). T1-weighted MRI showing bilateral globus pallidus hyperintensity, also resulting from iron overload.

**Figure 3.**
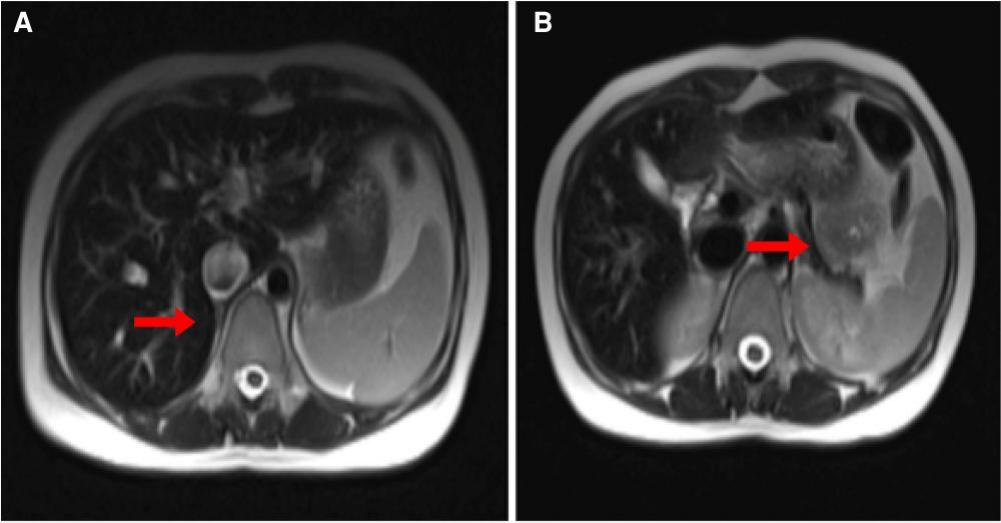
(A) Abdominal MRI showing hypointensity of the liver, suggesting iron deposition. (B) Abdominal MRI showing pancreatic hypointensity, consistent with pancreatic iron overload.

Imaging: Magnetic resonance imaging (MRI) of the brain revealed iron deposition: the pituitary gland was markedly hypointense on T2-weighted images, and bilateral globus pallidus regions showed T1 hyperintensity (Fig. 2, arrows), consistent with iron overload. Cardiac MRI demonstrated myocardial hypointensity indicating iron overload; echocardiography showed a normal left ventricular ejection fraction (60%). MRI of the liver and pancreas showed significant iron deposition (diffuse hypointensity on T2-weighted images) (Fig. 3).

Bone mineral density: Dual-energy X-ray absorptiometry (DEXA) confirmed osteoporosis: lumbar spine (L1−L4) T-score was −3.3 (*Z*-score −2.7), and femoral neck T-score was −3.7 (*Z*-score −3.1) (Table 2).

**Table 2.** Bone mineral density by DEXA scan

Skeletal site	T-score	*Z*-score	Normal T-score
Lumbar spine (L1-L4)	−3.3	−2.7	−1 to +1
Femoral neck (left)	−3.7	−3.1	−1 to +1
Femoral neck (right)	−3.5	−3.0	−1 to +1

T-scores and *Z*-scores, lowest values shown.

Abbreviation: DEXA, dual-energy X-ray absorptiometry.

This table presents DEXA scan findings, showing the lowest recorded T-scores and *Z*-scores for the lumbar spine and both femoral necks. T-scores are based on a young adult reference, whereas *Z*-scores are age-matched. All recorded values are in the osteoporotic range (T-score ≤ −2.5).

Genetic testing: Given the presentation, genetic analysis was performed. Next-generation sequencing revealed a homozygous missense variant in the *HJV* (hemojuvelin) gene exon 4: c.1063G > T (p.Asp355Tyr). This variant is extremely rare (not reported in major databases) and was classified as likely pathogenic. It confirmed the diagnosis of juvenile hemochromatosis type 2A due to *HJV* mutation.

## Treatment

The patient's management focused on reducing iron overload and addressing endocrine dysfunction. Therapeutic phlebotomy was initiated weekly, aiming to gradually reduce serum ferritin to approximately 50 ng/mL. For diabetes management, we used an intensive insulin regimen: ultra rapid-acting insulin lispro 5 to 10 units subcutaneously before each meal and long-acting insulin glargine 20 units at bedtime. Insulin doses were adjusted based on self-monitored blood glucose to improve glycemic control and to minimize hypoglycemia.

To treat hypogonadotropic hypogonadism and prevent estrogen-deficiency complications, we started cyclic hormone replacement: oral estradiol 2 mg daily with micronized progesterone 200 mg daily for 12 days each month. This regimen was intended to induce regular withdrawal bleeding and support bone health. For osteoporosis, she was prescribed oral calcium carbonate 1000 mg daily and cholecalciferol (vitamin D3) 2000 IU daily, with periodic monitoring of calcium and vitamin D levels. A bisphosphonate was considered but deferred given the initiation of estrogen therapy and young age and bone density will be reevaluated in follow-up. The patient was also advised on a diet low in iron and alcohol avoidance. Cardiology follow-up included repeat echocardiography every 3 months. Endocrinology follow-up included regular monitoring of endocrine function, ferritin levels, and glycemic status.

## Outcome and Follow-Up

After 3 months of weekly phlebotomy (8 sessions), the patient showed marked improvement. Her skin hyperpigmentation notably lightened and she experienced less fatigue. Glycemic control improved: Insulin requirements decreased (now lispro 5 units per meal, glargine 18 units nightly) with an HbA1c reduction from 8.5% to 7.8%. Serum ferritin fell from >2000 ng/mL to 1294 ng/mL (1.294 mg/L). Her hypogonadotropic state also improved: cyclic hormone therapy successfully induced menstrual periods by 2 months into treatment, and her most recent FSH and LH have risen slightly (FSH 5.2 mIU/mL, LH 2.6 mIU/mL). Importantly, estrogen replacement is expected to benefit her bone density in the long term.

Throughout therapy, no adverse events occurred. Repeat cardiac evaluation (MRI and echocardiogram) after 3 months showed stable cardiac iron levels and normal function (left ventricular ejection fraction 60%). Liver enzymes normalized (AST, ALT ∼25 IU/L). Her arthritis symptoms improved with lower ferritin and supplemental calcium/vitamin D.

The patient will continue maintenance phlebotomy (every 4-6 weeks) to keep ferritin <50 ng/mL, ongoing hormone therapy for bone protection, and a basal-bolus insulin regimen adjusted as needed. Emphasis is placed on long-term bone health: she has been counseled on bone-loading exercise and the need for specific antiosteoporotic therapeutic options if bone mineral density shows no further improvement on follow-up. Regular monitoring of iron studies, endocrine function, and bone mineral density is planned, given the risk of progressive organ damage in juvenile hemochromatosis.

## Discussion

Juvenile hemochromatosis (type 2A, *HJV*-related) is a life-threatening form of iron overload that presents in the second to third decade of life. Unlike classic HFE-hemochromatosis, which typically manifests after 40 years, JH manifests much earlier (often before age 30 years) and progresses rapidly [3]. The *HJV* gene encodes hemojuvelin, a co-receptor that upregulates hepcidin, the master iron hormone [2]. Loss-of-function *HJV* mutations cause near-complete hepcidin deficiency, leading to massive iron absorption and deposition in parenchymal tissues [2]. The pathophysiology of JH involves dysregulated hepcidin expression due to HJV gene mutations, leading to unregulated iron absorption from the gastrointestinal tract [5]. Excess iron accumulates in parenchymal tissues, including the liver, heart, pancreas, and pituitary gland, causing oxidative stress and organ dysfunction [6].

Our patient exhibited the classic features of JH: skin hyperpigmentation, endocrine failure (hypogonadism and diabetes), and osteopenia. Iron deposition in the pituitary causes gonadotropin deficiency and amenorrhea, as seen here with low LH, FSH, and estradiol [2]. Pancreatic iron causes β-cell loss and diabetes. Liver and cardiac iron overload can lead to cirrhosis and cardiomyopathy; fortunately, this patient had only mild liver enzyme elevation and preserved cardiac function, due to earlier intervention.

Diagnosing JH requires a comprehensive approach, including clinical evaluation, biochemical markers of iron overload, imaging, and genetic testing. Elevated serum ferritin (>2000 ng/mL) and transferrin saturation (>85%) that were present in our case are the hallmark biochemical findings of hemochromatosis [7]. The MRI findings of T2 pituitary hypointensity and T1 globus pallidus hyperintensity are characteristic of iron deposition in JH. We highlighted the globus pallidus in Fig. 2 to emphasize brain iron overload. Bone loss is also well-documented in hemochromatosis and may relate to both iron toxicity and hypogonadism [4]. Our patient's severe osteoporosis (T-scores < −3) underscores the need for aggressive bone-directed therapy; estrogen replacement in this context is expected to improve bone mineral density over time.

This case had a significant diagnostic delay (5 years of amenorrhea before diagnosis), a known issue in JH due to its rarity and nonspecific symptoms. The initial menstrual irregularity was misattributed to presumed polycystic ovary syndrome, and diabetes to type 2 diabetes. The combination of endocrine and arthritic symptoms eventually prompted a broader evaluation.

Management of JH hinges on early and intensive therapy. Phlebotomy is the cornerstone of management [8, 9] and regular weekly phlebotomies normalized this patient's iron stores and improved her symptoms. We also addressed secondary complications: insulin therapy for diabetes, hormone replacement for hypogonadism, and calcium/vitamin D for bone support. Our discussion emphasizes that multidisciplinary care is crucial, including various medical specialties like endocrinology, cardiology, and hepatology, as emphasized by Brissot et al, who also advocate for early diagnosis and aggressive iron removal to prevent irreversible organ damage [10].

In long-term follow-up, vigilant monitoring of bone health is required. Estrogen therapy and calcium/vitamin D supplementation address osteoporosis, but periodic DEXA scans and possible future bisphosphonate therapy or denosumab should be considered if bone density does not improve. Regular cardiac and liver surveillance is also indicated to detect any late cardiomyopathy or cirrhosis.

JH is a rare but serious disorder that should be considered in young patients with unexplained endocrine issues and systemic iron overload. Early diagnosis and aggressive management, including therapeutic phlebotomy, hormone replacement, and bone support, are key to preventing irreversible organ damage. Our patient's positive outcome highlights the benefits of timely, multidisciplinary intervention. Greater clinical awareness and prompt action can improve long-term outcomes and survival.

## Learning Points

- Suspect JH in young patients with unexplained hypogonadism, diabetes, or skin pigmentation; check iron studies early.
- Confirm diagnosis with genetic testing (eg, homozygous *HJV* mutation) to guide prognosis and family screening.
- Manage with regular phlebotomy and targeted therapies for endocrine and bone health.
- Address osteoporosis with hormone therapy and calcium/vitamin D; monitor with DEXA scans.
- Ensure multidisciplinary follow-up including cardiac and liver evaluation.

## Data Availability

Some or all datasets generated during and/or analyzed during the current study are not publicly available but are available from the corresponding author on reasonable request.
